# Five Years MIQE Guidelines: The Case of the Arabian Countries

**DOI:** 10.1371/journal.pone.0088266

**Published:** 2014-02-04

**Authors:** Afif M. Abdel Nour, Esam Azhar, Ghazi Damanhouri, Stephen A. Bustin

**Affiliations:** 1 Special Infectious Agents Unit-Biosafety Level 3, King Fahd Medical Research Center, King Abdulaziz University, Jeddah, Kingdom of Saudi Arabia; 2 Medical Laboratory Technology Department, Faculty of Applied Medical Sciences, King Abdulaziz University, Jeddah, Kingdom of Saudi Arabia; 3 King Fahd Medical Research Center, King Abdulaziz University, Jeddah, Kingdom of Saudi Arabia; 4 Postgraduate Medical Institute, Faculty of Health, Social Care & Education, Anglia Ruskin University, Cambridge, United Kingdom; University of California, Irvine, United States of America

## Abstract

The quantitative real time polymerase chain reaction (qPCR) has become a key molecular enabling technology with an immense range of research, clinical, forensic as well as diagnostic applications. Its relatively moderate instrumentation and reagent requirements have led to its adoption by numerous laboratories, including those located in the Arabian world, where qPCR, which targets DNA, and reverse transcription qPCR (RT-qPCR), which targets RNA, are widely used for region-specific biotechnology, agricultural and human genetic studies. However, it has become increasingly apparent that there are significant problems with both the quality of qPCR-based data as well as the transparency of reporting. This realisation led to the publication of the Minimum Information for Publication of Quantitative Real-Time PCR Experiments (MIQE) guidelines in 2009 and their more widespread adoption in the last couple of years. An analysis of the performance of biomedical research in the Arabian world between 2001–2005 suggests that the Arabian world is producing fewer biomedical publications of lower quality than other Middle Eastern countries. Hence we have analysed specifically the quality of RT-qPCR-based peer-reviewed papers published since 2009 from Arabian researchers using a bespoke iOS/Android app developed by one of the authors. Our results show that compliance with 15 essential MIQE criteria was low (median of 40%, range 0–93%) and few details on RNA quality controls (22% compliance), assays design (12%), RT strategies (32%), amplification efficiencies (30%) and the normalisation process (3%). These data indicate that one of the reasons for the poor performance of Arabian world biomedical research may be the low standard of any supporting qPCR experiments and identify which aspects of qPCR experiments require significant improvements.

## Introduction

The last few years have witnessed a significant growth in applications for relatively high-throughput techniques such as real time quantitative PCR (qPCR), microarray analysis and Next Generation Sequencing (NGS). qPCR in particular has become a ubiquitous molecular technology, mainly due to its perceived simplicity, sensitivity, speed and low cost. A “Web of Knowledge” search using the term “real-time PCR” records the use of this technique in 174,295 publications between 2004 and 2012 in comparison to only 18,065 articles between 1993 and 2003. Not surprisingly, this popularity has resulted in a wide range of different protocols, instruments, assay designs and analysis methods that have resulted in the publication of data that are often contradictory and not reproducible[1]. This was the subject of an editorial in BMC Molecular Biology[2] and was recently taken up in more general terms in a Nature editorial, which posited that one of the main problems with data reproducibility is the lack of scrutiny afforded to the technical detail of publications[3].

Consequently there has been a growing consensus around the need to improve the transparency of reporting of relevant experimental detail to include every aspect important to the qPCR assay itself, as well as issues relating to pre- and post-assay parameters. This awareness resulted in the publication of the MIQE guidelines[4] in 2009, with a follow-up publication proposing guidelines for digital PCR published earlier this year[5]. These provide a set of recommendations that can be used by journal reviewers to help them evaluate the reliability of a publication's experimental protocols and ensure the inclusion of all essential technical information in the final publication. Five years after their publication, the research community is now beginning to embrace these guidelines, with nearly 2,000 citations recorded by December 2013 and a recent comparison of publications shows a significantly improved standard of reporting in papers that cite the guidelines compared with those that do not[6]. Nevertheless, it is also important to state that citation of the MIQE publication does not guarantee actual observance of the guidelines[7] and that the vast majority of reverse transcription (RT)-qPCR publications do not comply with even the most basic reporting guidelines[6]. To help with compliance, an iOS/Android app has been developed for mobile devices, tablet and Personal Computers[8], with major suppliers providing extensive online advice and checklists to assist their customers with MIQE compliance (for example http://www.roche-applied-science.com/campaigns/MIQE/).

According to a 2010 report by the UNESCO, the landscape of Research and Development in the Arabian countries is positively changing with an increase of almost 45% in the number of scientific research articles from 2000 to 2008[9]. Several Arabian countries, especially the Kingdom of Saudi Arabia, Qatar and the United Arab Emirates, are commissioning cutting-edge research facilities second to none, for example the Sidra Medical and Research Center, a world class multi-billion hospital and health research institute in Qatar or the King Abdulaziz University and King Abdullah University of Science and Technology in the Kingdom of Saudi Arabia and several prestigious American and European universities have developed a presence in the United Arab Emirates. At the same time an analysis of the performance of biomedical research in the Arabian world during 2001–2005 suggests that the Arabian world is producing fewer biomedical publications, which are of lower quality than those from other Middle Eastern countries[10].

The aim of this analysis was to evaluate the transparency of reporting of technical detail in peer-reviewed papers published between 2009 and 2013 that utilised RT-qPCR from the 22 countries of the Arabian league and evaluate whether the technical standards of these publications has improved.

## Methods

**Figure 1:**
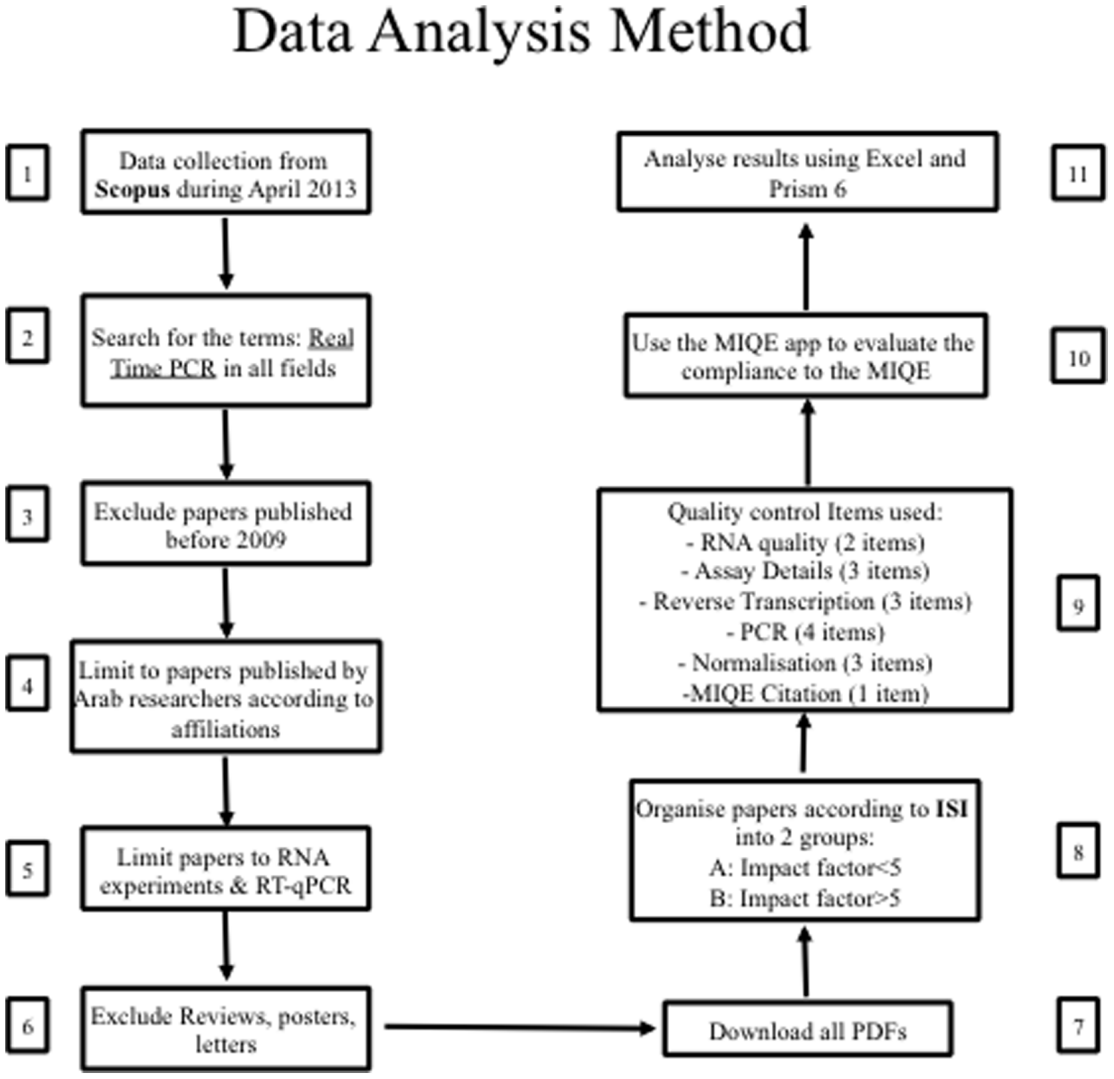
Data analysis method. Eleven steps needed for the analysis of 461 scientific papers related to RT-qPCR.

RT-qPCR-related articles published by the Arabian countries were identified as follows (Fig. 1):

- The Scopus database was screened for the terms “Real time PCR”, with all papers included that were published between 2009 and 2013 by authors affiliated to any one of 22 countries forming the Arabian league. Only articles targeting cellular RNA were used in the analysis, excluding those targeting viral RNA.
- Quality and compliance with the MIQE guideline were analysed based on the criteria shown in Table 1: RNA quality (two parameters), assays design (three parameters), RT strategies (three parameters), amplification details (four parameters) and normalisation (two parameters+ number of reference genes). Our focus on these 15 criteria, which include the most critical MIQE parameters, was done for practical reasons to minimize complexity. They do not imply that the others can be neglected.
- Parameters were recorded as compliant (“1”) or non-compliant (“0”). To calculate compliance for each publication, individual compliance scores were added to give a maximum value of 15 (100% compliance). Overall compliance was determined by calculating the median of these individual compliance values.
- Compliance for individual parameters was obtained by adding the scores for the individual publications making up categories A (impact factor (IF) <5) or B (IF≥5) and expressing them as a percentage.
- The MIQE app was used to simplify the data collection and all data were analysed using a Microsoft Excel spreadsheet (office.microsoft.com). Results are expressed as a percentage of compliance with individual MIQE criteria. Normalisation was evaluated by noting the number of reference genes.
- Publications appearing after 2011 were further analysed to determine whether the quality of the data and transparency of reporting differed from earlier publications where the authors might not have been expected to be aware of the MIQE guidelines. All statistical analyses were carried out using GraphPad Prism version 6.00 for Mac OS X (GraphPad Software, San Diego California USA, www.graphpad.com). Data were tested to determine whether they came from a normal distribution and appropriate statistical tests were used for analysis.

**Table 1:** Quality and compliance with MIQE guideline analysed criteria.

Items	Analysed Parameters	Method of analysis
**Journal Name**	Impact factor	IF is just for information; do not aim for high or low, just for journals that are of interest to you
	PubMed ID number	NCBI website
	Online supplement	‘yes’ if online supplemental file(s) is available
**RNA Quality**	Cellular RNA	for this survey we do not want to look at viral RNA
	RNA purity	‘yes’ if there is any assessment of purity, through e.g. inhibition assay (SPUD or alike), target and sample-specific dilution curve, global UV-VIS absorption spectrum, …
	RNA integrity	‘yes’, if there is any assessment of integrity, such as microfluidic electrophoresis (Experion, Bioanalyser, or alike), gel electrophoresis, 5′-3′ assay, …
**Assay details**	Primer (probe) sequences/assay ID	‘yes’ if primer (and probe) sequences are provided
	PCR efficiency	‘yes’ if there is any assessment of amplification efficiency
	Assay specificity	‘yes’ if there any mentioning of in silico homology search (BLAST, ePCR, BiSearch, or alike), amplicon sequencing, restriction digest, amplicon length determination, melting curve, …
**Reverse transcription**	Input amount of RNA in RT reaction	‘yes’ if input amount of RNA in RT reaction is mentioned (also see below)
	RT enzyme or RT kit	‘yes’ if there is any mentioning of reverse transcriptase used or specific kit, along with minimal instructions (can be according to manufacturer)
	priming method	‘yes’ if type of primers are mentioned (random primers, oligo-dT, blend, gene specific primers, …)
**PCR**	PCR conditions	‘yes’ if PCR conditions are listed or referred to an older publication
	Taq polymerase or PCR kit	‘yes’ if there is any mentioning of Taq polymerase used or specific kit, along with minimal instructions (can be according to manufacturer)
	Final primer concentration	‘yes’ if final primer concentration in reaction is mentioned (or can be deduced)
	Input amount template in PCR reaction	‘yes’ if input amount of template is mentioned; cDNA concentration does not have to be measured, can be RNA equivalents (e.g. 1 µg of total RNA is reverse transcribed in a 2-step reaction in 20 µl; 1/10 is used for PCR, which means 5 ng total RNA equivalents gets into PCR reaction)
**Normalisation**	More than 1 reference gene	‘yes’ if more than one reference gene is used
	If yes: number of reference genes	
	Reference gene validation	‘yes’ if there is any indication of reference gene validation method (e.g. geNorm or alike; can also be referral to previous paper in which their expression stability was validated in similar experimental conditions)
**MIQE**	Citing the original MIQE	Citing the original MIQE paper (Clinical Chemistry 2009)

## Results and Discussion

**Figure 2:**
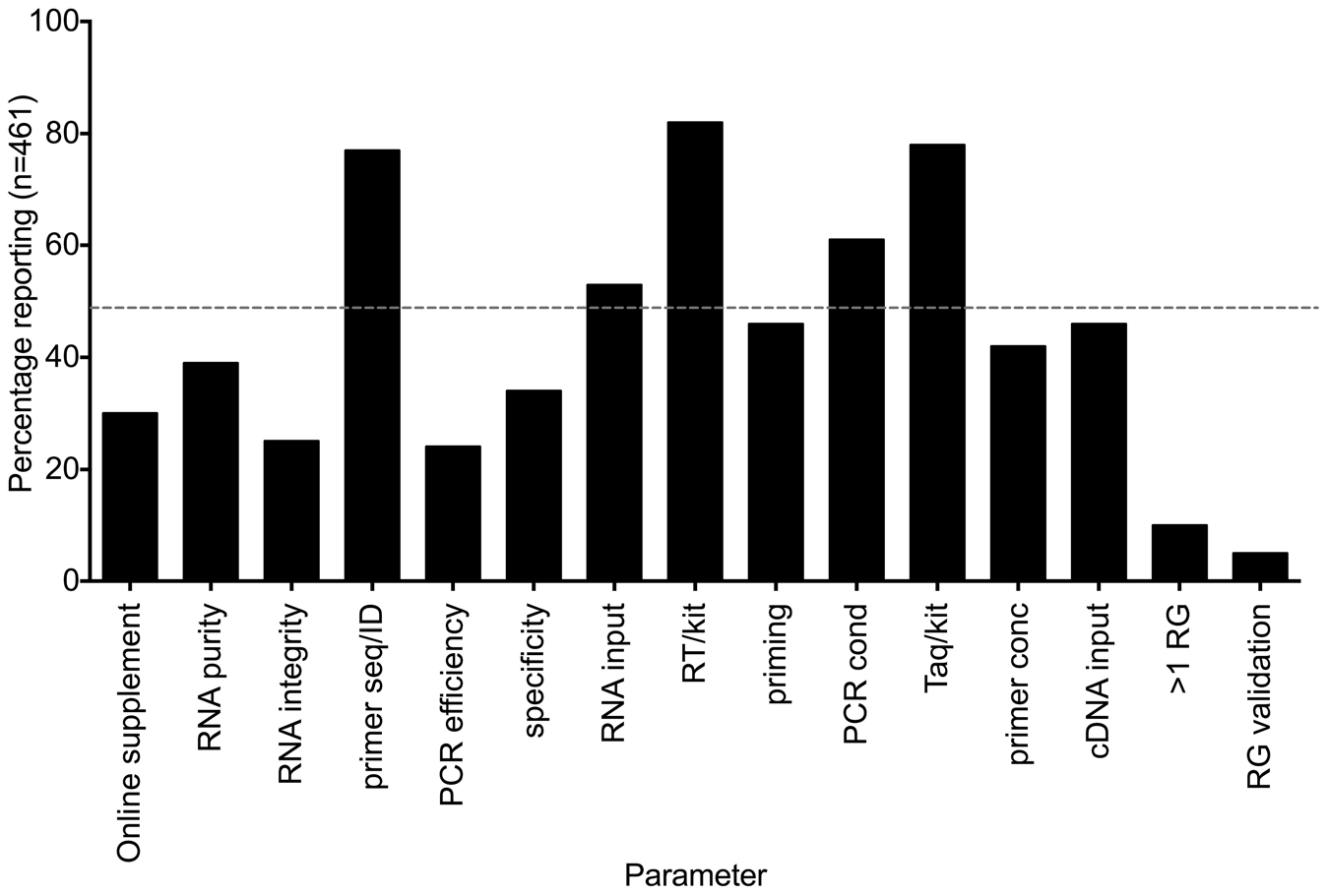
% compliance with 15 MIQE parameters by all 461 publications between 2009 and 2013. The hatched grey line indicates 50% compliance.

Transparency of reporting of materials and methods is critical for reproducibility of RT-qPCR-based experiments, which are made up of a complex series of steps that remain inadequately standardised[1]. Hence 461 articles were scrutinized for their compliance with 15 critical parameters. Overall compliance with the MIQE guidelines was low, with a median of six out of 15 parameters being reported (range 0-14). There was no significant difference in compliance with the guidelines between different regions comprising African Arabian Countries (Algeria, Egypt, Libya, Morocco, Sudan, and Tunisia), the Kingdom of Saudi Arabia and the Gulf States/Middle East (Bahrain, Iraq, Jordan, Kuwait, Lebanon, Oman, Palestine, Qatar and Syria) (Kruskall-Wallis test, p = 0.424). The recording of RNA integrity, PCR efficiency and reference gene data was especially inadequate (Figure 2).

Reporting of RNA quality should involve an assessment of both its purity (absence of inhibitors) and integrity; only 39% of publications reported RNA purity and 25% RNA integrity. The purity data flatter, since they include assessment by spectrophotometer, which provides no information on the presence of potential inhibitors in an RNA preparation. Instead, a comparison of diluted samples or inclusion of an inhibition control such as the SPUD assay[11] is advisable. Information about RNA integrity is also essential since it directly affects the Cq valued recorded by a sample[12].

An analysis of the assay design criteria, which comprise three parameters (Table 1) showed that 77% of the papers provided either the primer sequences or the assay of commercial assays, thus complying with the modified MIQE criteria[13]. Unfortunately, PCR efficiency and assay specificity were characterised by inadequate reporting at 24% and 34%, respectively. Given the importance of comparing qPCR assays of matching amplification efficiency and ensuring their specificity, this is unacceptably low[14].

RT and PCR conditions, represented here by seven parameters, have a significant impact on cDNA yield and levels of mRNA expression[15]. Reporting of these parameters was somewhat higher, although still far from universal and is probably explained by the fact that their reporting involves no additional validation work on the part of the authors.

Appropriate normalisation is essential for reliable and biologically meaningful reporting of RNA expression levels. This requires the selection of multiple reference genes that have been properly validated[16]. Unfortunately, the vast majority of publications use a single reference gene that has not been validated: only 29 papers normalised the expression of their genes of interest to two genes, and only 15 papers used more than two genes. This is very likely to result in conclusions that are not supported by the actual results but are based on artifacts due to the inadequate and inappropriate normalisation process[17].

**Figure 3:**
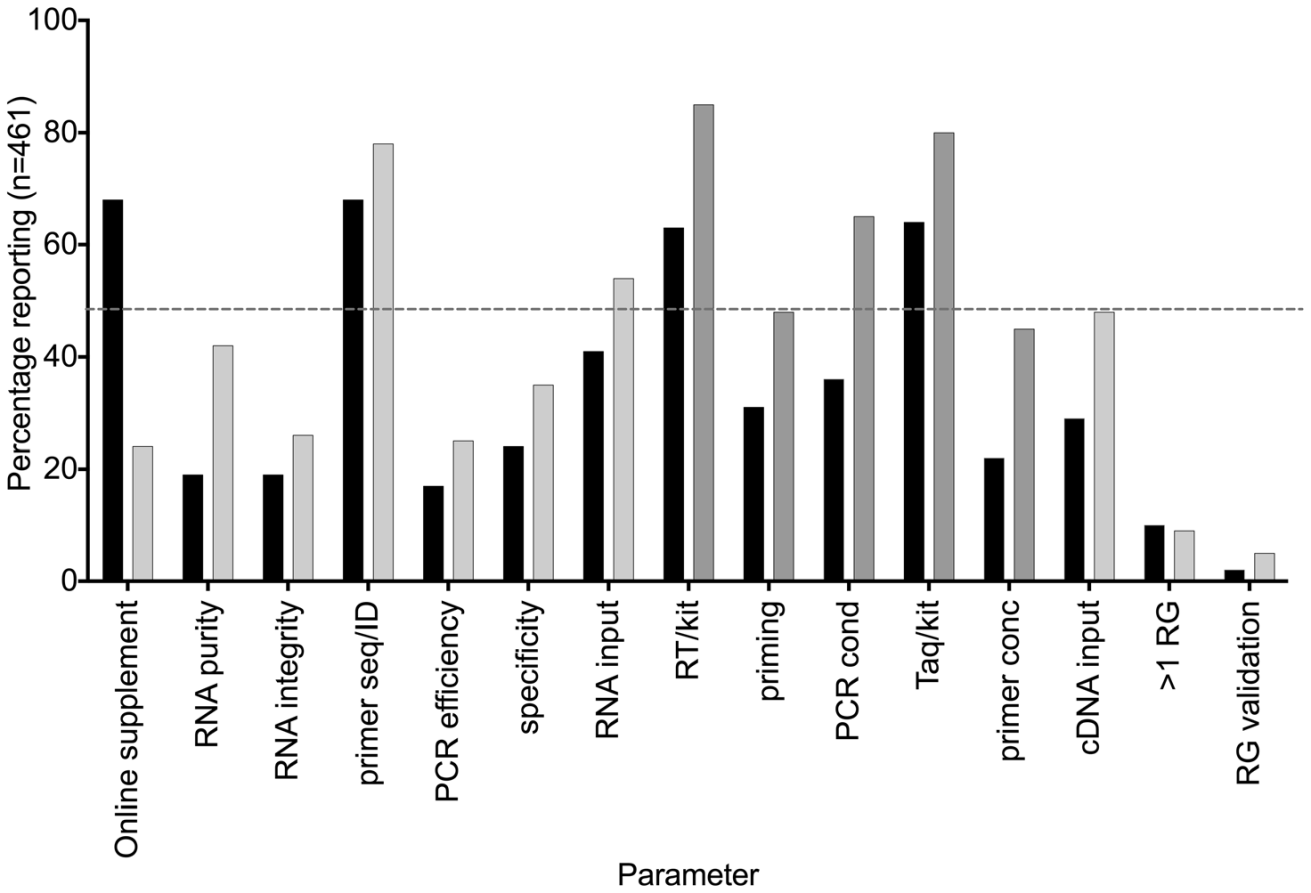
Comparison of online supplement utilization and MIQE compliance between publications with IF<5 (grey) and those ≥5 (black). Both datasets passed the D'Agostino & Pearson and Shapiro&Wilks normality tests, hence the parametric paired t-test was used for data analysis). The hatched grey line indicates 50% compliance.

**Figure 4:**
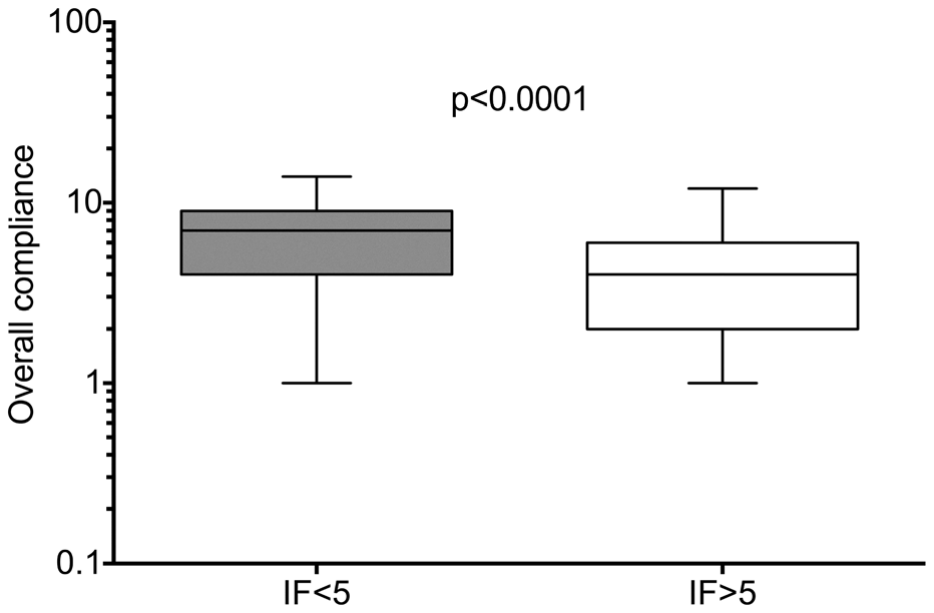
Overall compliance with MIQE guidelines of category A papers (journals with IF<5, n = 402) compared to category B papers (journals with IF>5, n = 59). Neither dataset passed the D'Agostino & Pearson and Shapiro&Wilks normality tests, hence the nonparametric Mann-Whitney test was used for data analysis).

**Figure 5:**
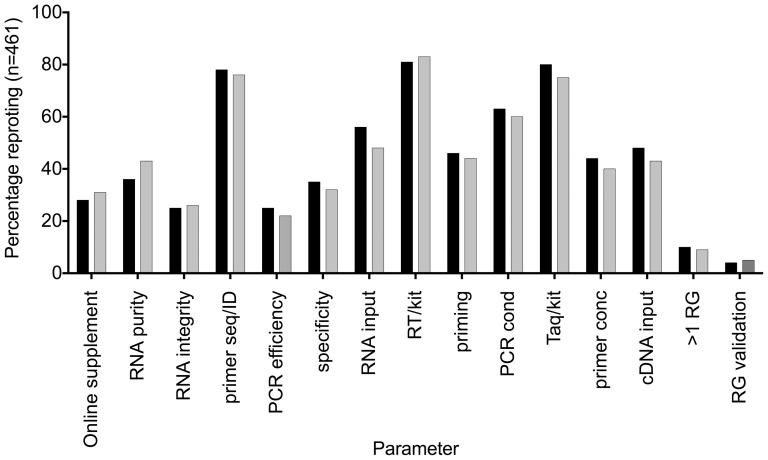
% compliance with the 15 MIQE parameters of papers published up to 2011 (black) and from 2011–2013 (grey).

Articles were stratified according to their journal's IF and divided into two categories, those published in journals with no IF or an IF of <5 (category A, n = 402) and those with IFs of 5 or above (category B, n = 59). Although only 24% of papers published in category A journals make use of online supplements, compared with 68% of those in category B, they report significantly more experimental detail (Figure 3, paired t-test p = 0.034), resulting in a negative correlation between IF and MIQE compliance (Spearman r = −0.212, CI −0.3 to −1.12), p<0.0001). A comparison of overall compliance stratified according to IF shows that publications in journals with IF<5 are significantly more compliant than those in journals with IF ≥5 (Figure 4, Mann-Whitney p<0.0001). Finally, we compared publications that appeared between 2009 and 2011 with those from 2012/13, to determine whether there was any improvement in transparency of reporting. Figure 5 shows that there is no such improvement (Mann-Whitney p = 0.798), which suggests either that the vast majority of Arabian authors are unaware of the existence of these guidelines or chooses to ignore them.

Only eight publications (2%) cited the 2009 MIQE paper, seven of which were published in journals with IFs<5. Their standard of reporting was significantly better than that of those not citing the MIQE guidelines, with a median compliance of 73% (range 60–93%, Mann-Whitney p<0.001). However, even here only 2 papers (25%) validated or made use of two or more reference genes.

## Conclusions

We conclude that MIQE awareness in Arabian countries is very poor and has not improved since 2011, when one might have expected researchers to become familiar with the concept. This suggests that much work still needs to be done by Arabian researchers to implement the transparency criteria advocated by MIQE guideline. An interesting application of the MIQE guideline to large-scale clinical and pre-clinical trials showed that although there was an increase of 4–7% in the cost of qPCR experiment, no additional time or manpower were needed to follow the MIQE guideline[18]. Given that the results are likely to be more reliable, reproducible and clinically relevant, this seems an appropriate price to pay for better quality data.
